# Acetylation/methylation at lysine 9 in histone H3 as a mark of nucleosome asymmetry in human somatic breast cells

**DOI:** 10.1038/s41420-020-0278-z

**Published:** 2020-05-26

**Authors:** Bruno Perillo, Annalisa Di Santi, Gustavo Cernera, Giovanni Galasso, Gabriella Pocsfalvi, Gabriella Castoria, Antimo Migliaccio

**Affiliations:** 1 Istituto per l’Endocrinologia e l’Oncologia Sperimentale “G. Salvatore”, C.N.R, 80131 Naples, Italy; 2 Azienda Ospedaliera dei Colli, 80131 Naples, Italy; 3 Dipartimento di Medicina Molecolare e Biotecnologie Mediche, Università di Napoli “Federico II”, 80131 Naples, Italy; 4 Dipartimento di Medicina di Precisione, Università della Campania “L. Vanvitelli”, 80138 Naples, Italy; 5 Institute of Biosciences and Bioresources, 80131 Naples, Italy

**Keywords:** Histone post-translational modifications, Epigenetics

Nucleosomes, the basic structural units of chromatin, consist of a 147 bp DNA fragment wrapped around a protein octamer containing two copies each of histones H2A, H2B, H3, and H4. Histones exhibit a variety of posttranslational modifications (PTMs) that are recognized by multimeric effector proteins and act in concert to control gene expression^1,2^. Deep work has been made in the last years to highlight the combinations of histone marks that concomitantly occur within a nucleosome, with the new front-line focused to evidence whether a specific modification is present on one or on both residues of each histone pair. Accordingly, growing evidence has accumulated on the existence and function of the so-called nucleosomal “bivalent domains” where activating and repressive PTMs added on different residues coexist at the promoters of developmentally regulated genes in embryonic stem cells that resolve this apparently contradictory pattern upon differentiation^3–5^.

We asked whether asymmetric nucleosomes are present also in somatic cells and pointed our attention on one of the best characterized inhibitory PTMs, trimethylated lysine 9 in histone H3 (H3K9me3)^6^. This residue is peculiar, as it can be also subjected to acetylation, behaving, under this condition, as an activating signal for transcription^7^. Therefore, we questioned whether nucleosomes at the promoters of inducible genes showed, when not stimulated, both lysines methylated or if one residue was unmodified or, more interestingly, acetylated, presumably as an outcome of previous transcriptional activation. To address this issue, we have analyzed by chromatin immunoprecipitation (ChIP) the promoter of pS2 gene in the human breast MCF-7 cells, that is not transcribed in the absence of estrogen (E2) and can be activated by hormone addition to the culture medium^8^.

**Fig. 1:**
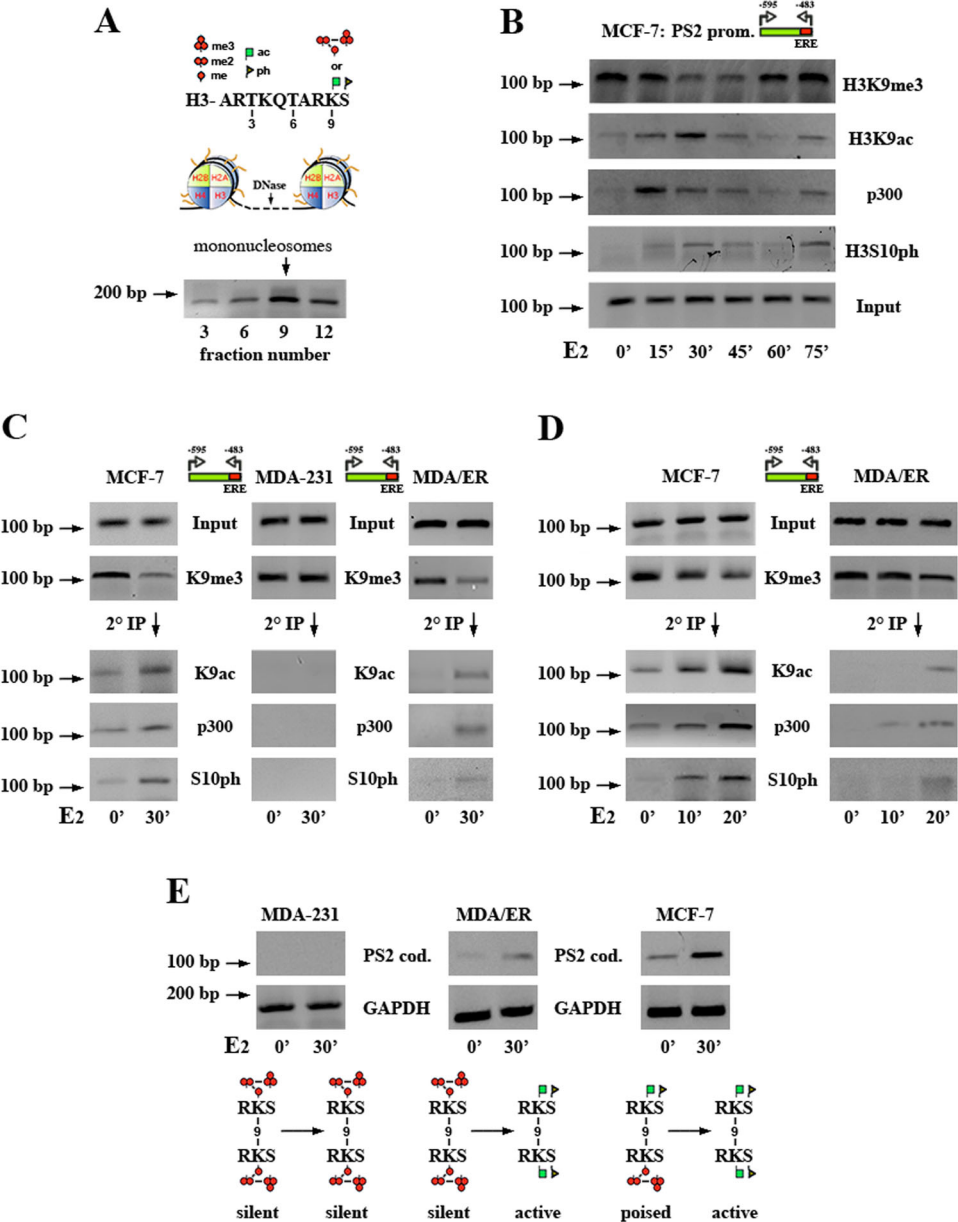
**a** Highlighting nucleosome asymmetry at promoters of poised genes. Graphic representation of PTMs at the N-terminal tail in histone H3. In the middle, two adjacent nucleosomes subjected to digestion with micrococcal nuclease (DNase) to yield mononucleosomes selectively collected by sucrose gradient centrifugation. At the bottom, a representative electrophoretic gel where the vertical arrow points to the fraction from sucrose gradient with the highest percentage of monomeric nucleosomes chosen to be further processed in ChIP assays, according to established procedures^5^. The same approach has been followed for all digested chromatin samples. The horizontal arrow on the left (as in the following sections) indicates the molecular weight expressed as base pairs length. **b** ChIPs from MCF-7 cells where the estrogen-responsive promoter of pS2 gene was amplified after capture with the antibodies reported on the right of one descriptive gel, according to previously reported protocols^9^. ERE is the estrogen responsive element present in pS2 promoter. Cells were starved from E2 and then challenged with the hormone for different times. **c** Re-IPs where immunoprecipitated chromatin from MCF-7 or ERα^−^ MDA-231 breast cells expressing (MDA/ER, right) or not (MDA-231, middle) exogenous receptor was incubated with a second antibody, as already reported^9^, to establish whether H3K9me3 and H3K9ac followed the combinatorial model. **d** The same Re-IPs as in section **c**, realized to compare the time of H3K9ac and p300 appearance in hormone-starved MCF-7 and in MDA/ER cells. **e** Assessment of pS2 transcription by RT-PCR. Below each gel, the “transcriptional status” of pS2 gene in the absence or presence of E2 has been graphically reported.

ChIP is one of the most used experimental approaches to evidence epigenetic modifications on a specific chromatin locus but it cannot discriminate if a mark is present on one or on both tails of twin histones within a nucleosome, even though searching for modifications that targeted the same residue (H3K9) should work as a more unambiguous approach. As a first step, to avoid the stabilization of any looping, we digested native (not cross-linked) chromatin with micrococcal nuclease (DNase) to generate mononucleosomes that were, then, purified by sucrose gradient in order to collect excluysively mononucleosomes^5^ (Fig. 1a). As shown in Fig. 1b, between 0 and 30 min of E2 challenge we observed the expected cyclic behavior of estrogen-induced PTMs^9^, with a decrease of H3K9me3 and a concomitant increase of acetylated lysine 9 (H3K9ac) at pS2 promoter, also accompanied by targeting of the p300 histone acetyltransferase (HAT)^10^ and appearance of phosphorylated serine 10 in the same histone (H3S10ph), a mark that labels active genes^11^. Reported ChIP assays revealed that pS2 promoter was occupied by nucleosomes with H3 histones where lysine 9 was either methylated and acetylated, even though with changing percentages throughout the examined time-course. Therefore, to be sure that we were observing bivalent nucleosomes and to exclude that each lysine 9 was differently marked in any of the two allelic pS2 genes, we performed sequential ChIP (Re-IP) experiments by reimmunoprecipitating H3K9me3 immune-complexes with antibodies to H3K9ac. Our results supported the combinatorial model in which acetylation, gained through recruitment of p300, and trimethylation of K9 were present within the same nucleosome that also harbored H3S10ph (Fig. 1c, left).

Based on these data, we hypothesized that pS2 promoter hosted an asymmetric nucleosome and that the gene could be considered as a poised gene where, in the absence of stimulatory signals, acetylation of H3K9 on one histone should represent a barrier to full trimethylation, presumably obtained by inhibiting recruitment of histone methyltransferases (HMTs) while enhancing that of HATs^12^. Interestingly, the same nucleosome showed an H3S10ph whose presence has already been reported to prevent methylation of the preceding lysine^13^. We, then, speculated that asymmetry emerged after the first cycles of gene expression and, to test this hypothesis, we analyzed the estrogen receptor α^−^ (ERα^−^) breast MDA-231 cells that, being unable to respond to E2 challenge, should lack histone asymmetry at the pS2 promoter. As predicted, these cells, even under estrogen stimulation, showed a stable level of H3K9me3 and absence of H3K9ac, with the switch (together with targeting of p300 and appearance of H3S10ph) generated exclusively after internalization of exogenous ERα by transfection (Fig. 1c, middle and right).

As a further step, we compared the behavior of the asymmetric nucleosome at pS2 promoter in MCF-7 and ERα-transfected MDA cells at early times after E2 addition. While nucleosomes captured with antibodies to H3K9me3 from MCF-7 cells revealed their bivalency even in the absence of E2 with one of the two lysines 9 already acetylated, those captured from transfected MDA (MDA/ER) cells, showed the appearance of H3K9ac and H3S10ph, a mark, by the way, whose pre-requisite is demethylation of the preceding residue^14^, only after 20 min of E2 challenge (Fig. 1d). In fact, transcriptional activation of pS2 gene, absent in wild-type MDA, was delayed in MDA/ER cells (Fig. 1e).

According to our data, we propose the following model: when the estrogen receptor triggers the first transcriptional cycle, it induces demethylation of both lysines 9, a condition permissive for assembly of the transcriptional machinery. This step is followed by phosphorylation of serine 10 in the same histone that acts as a barrier to fast remethylation^14^. Successively, one of the two serines is dephosphorylated and the adjacent lysine can be, thus, remethylated, representing the basis for next cycles of demethylation by the specific demethylases with oxidation of nearby guanines that allow transcription^6^. We speculate that, leaving only one of two lysines 9 to be demethylated helps the machinery triggered by recruitment of ERα to find the right direction for transcription, as oxidation of guanines has been reported to have consequences also on DNA methylation in a strand-specific manner^15^.

In this study, we show the first example of histone asymmetry affecting the same residue subjected to modifications with an inverse effect on gene expression. Interestingly, the involved residue is the one that drives estrogen-induced transcription through generation of reactive oxygen species (ROS) triggered by its demethylation^6^. This model appears to be not restricted to the reported estrogen-responsive gene, as we have evidences that a very similar picture is present on the paradigmatic retinoic acid target gene cyp26A1. Incidentally, we remind that the two hormones have the opposite effect on cellular life and, then, we can imagine this as a more general phenomenon, by which cells should be able to respond quickly to cognate stimuli. We are aware that our data need further investigation at a genome-wide level and deeper analysis of the molecular mechanism elicited; however, we are persuaded that highlighting the existence of somatic nucleosomal asymmetry that, in particular, is characterized by two opposite marks deposited onto the same residue, could be significant per se.
